# Preparation of Functionalized Magnetic Fe_3_O_4_@Au@polydopamine Nanocomposites and Their Application for Copper(II) Removal

**DOI:** 10.3390/polym10060570

**Published:** 2018-05-23

**Authors:** Yanxia Li, Lu Huang, Wenxuan He, Yiting Chen, Benyong Lou

**Affiliations:** Department of Chemical Engineering and Materials, Ocean College, Minjiang University, Fuzhou 350108, China; 1991@mju.edu.cn (L.H.); 2239@mju.edu.cn (W.H.); cyt@mju.edu.cn (Y.C.); lby@mju.edu.cn (B.L.)

**Keywords:** magnetic nanocomposites, copper(II), polydopamine, Fe_3_O_4_@Au nanoparticles

## Abstract

Polydopamine (PDA) displays many striking properties of naturally occurring melanin in optics, electricity, and biocompatibility. Another valuable feature of polydopamine lies in its chemical structure that incorporates many functional groups such as amine, catechol and imine. In this study, a nanocomposite of magnetic Fe_3_O_4_@Au@polydopamine nanopaticles (Fe_3_O_4_@Au@ PDA MNPs) was synthesized. Carboxyl functionalized Fe_3_O_4_@Au nanoparticles (NPs) were successfully embedded in a layer of PDA through dopamine oxypolymerization in alkaline solution. Through the investigation of adsorption behavior to Cu(II), combined with high sensitive electrochemical detection, the as-prepared magnetic nanocomposites (MNPs) have been successfully applied in the separation and analysis of Cu(II). The experimental parameters of temperature, Cu(II) concentration and pH were optimized. Results showed that the as-prepared MNPs can reach saturation adsorption after adsorbing 2 h in neutral environment. Furthermore, the as-prepared MNPs can be easily regenerated by temperature control and exhibits a good selectivity compared to other metal ions. The prepared Fe_3_O_4_@Au@PDA MNPs are expected to act as a kind of adsorbent for Cu(II) deep removal from contaminated waters.

## 1. Introduction

It is important that freshwater be free from toxic chemicals for industry, agriculture and human health [1]. The contamination of toxic heavy metals in aqueous systems brings serious threats to the environment, even though a trace intake of different metals is key for human health [2]. These metals are in the form of ions that interact with proteins, nucleic acids and other biological ligands to form metal proteins, metal enzymes and other biological complexes and also play an important biochemical and physiological role in the life process. These metals might lead to morbidity if the content of metals in the human body is too high or too low [3]. The development of technologies for water purification is critical to meet the global challenges of insufficient water supply and inadequate sanitation. Among all treatments for heavy metals, adsorption is globally recognized as a very attractive technique because of its simplicity, reversibility and economic feasibility [3,4]. Therefore, development of novel materials as adsorbents for removing heavy metals from wastewater has been widely addressed [5,6].

Copper ions play an important role in many areas, such as chemical, biological and environmental fields. However, excessive intake of copper ions produces severe toxicological effects, such as nausea, diarrhea, vomiting, stomach cramps, or even death. Along with the extensive use of copper in industry, copper contamination is an important environmental problem and has attracted more and more attention [7,8,9]. The World Health Organization recommends the maximum limit of Cu ions in drinking water as 1.5 mg/L, whereas the US EPA defines it as 1.3 mg/L [10,11]. Therefore, effective removal of concentrated copper from industrial and agricultural wastewater is of fundamental importance.

There are many reports on Cu(II) removal from wastewater with natural, modified, or composite adsorbents [12,13,14,15,16]. Datta et al. used rioctylamine supported sontmorillonite for adsorptive separation of Cu(II) from an aqueous solution [12]. Dichiara et al. describes the aqueous-phase adsorption of Cu(II) on free-standing hybrid papers comprised of both graphene and single-wall CNTs [13]. These composite materials show great promise for separation and enrichment, environmental administration and wastewater treatments. The intrinsic properties of materials and the physicochemical parameters such as the pH of aqueous solution, initial metal ion concentration, and time can affect the adsorption of copper ions. Environmentally friendly materials with higher adsorption capacity, higher selectivity, and that are more cost-efficient are in urgent need.

Recently, various inorganic nanomaterials as carriers, such as iron oxide, carbon nanotubes, metal oxide, etc., have been applied for removal of metal ions through surface modification [4]. Among these nanostructured materials, core-shell magnetic materials have been widely favored by domestic and overseas researchers owing to their outstanding performance [17]. Firstly, these magnetic materials can be easily removed from the system by an external magnetic field. Secondly, they are cheap and easy to synthesize. Finally, they can be functionalized with various chemical species on the particle surface to potentiate the specific affinities to metal ions [15,18,19].

In modern materials science, surface coatings and modifications allow control of the surface properties to confer new functionalities for them. Polydopamine displays many striking properties of naturally occurring melanin in optics, electricity, and magnetics, and biocompatibility. Another valuable feature of polydopamine lies in its chemical structure that incorporates many functional groups such as catechol, amine, and imine. These functional groups can serve as both the starting points for covalent modification with desired molecules and the anchors for the loading of transition metal ions [20]. In fact, previous literature reported that magnetic graphene@polydopamine composites exert excellent adsorption efficiency to Cu(II) and were applied to enrich and identify low concentration standard peptides [21]. This phenomenon indicates that PDA can effectively combine with copper ions. In addition, PDA exhibits potential to immobilize metal ions (e.g., Ti^4+^, Fe^3+^, Cu^2+^) [22,23]. In the present study, novel magnetic Fe_3_O_4_@Au@PDA nanocomposites were synthesized. The properties of the composites were investigated in detail. Through the investigation of adsorption behavior to Cu(II), combined with high sensitive electrochemical detection, the prepared Fe_3_O_4_@Au@PDA MNPs have been successfully applied in the separation and analysis of copper ions. Results demonstrate the great potential of the composites for versatile water purification and treatment.

## 2. Experimental Section

### 2.1. Materials

CuCl_2_, AgNO_3_, Mn(NO_3_)_2_, MgCl_2_, NiCl_2_·6H_2_O, CdCl_2_, Pb(NO_3_)_2_, FeCl_3_·6H_2_O, FeCl_2_·4H_2_O, 25–28% NH_3_·H_2_O, HCl, NaOH, KCl, sodium citrate, gold chloride tetrahydrate (HAuCl_4_·4H_2_O), dopamine hydrochloride (DA·HCl, 98%), mercaptopropionic acid (MPA) of analytical grade were received from Fuzhou Xinyuhua Experimental Instrument Co., Ltd. (Fuzhou, China), and the solvent is deionized water.

### 2.2. Preparation of Fe_3_O_4_@Au-COOH NPs

The sodium citrate dispersed Fe_3_O_4_ NPs were prepared according to the published literature [24]. Sodium citrate (0.069 g) was then added to 40 mL of dispersed Fe_3_O_4_ NPs under vigorous stirring. A total of 32 mg gold chloride tetrahydrate was added rapidly and continued under reflux for 30 min before being allowed to cool to normal atmospheric temperature. The resulting colloidal solution was dissolved with 1 mol/L HCl to remove unwrapped Fe_3_O_4_ NPs, then isolated in a magnetic field to remove independent Au NPs and washed several times with water until the solution is neutral. The resulting Fe_3_O_4_@Au colloidal solution (Fe_3_O_4_@Au) in 40 mL deionized water was added to 0.5 mL of MPA stirred for 30 min, and washed several times with deionized water, then dried at 60 °C overnight, producing MPA modified Fe_3_O_4_@Au NPs (Fe_3_O_4_@Au-COOH NPs).

### 2.3. Preparation of Fe_3_O_4_@Au@PDA MNPs

First, 20 mg of dopamine was dissolved in the suspension of Fe_3_O_4_@Au-COOH NPs (30 mg) and dispersed by ultrasound in a 5 mL buffer solution (10 mmol/L Tris, pH = 8.5). The mixture was shaken at room temperature. After the reaction, the Fe_3_O_4_@Au@PDA MNPs were collected by magnetic separation and washed with water several times to remove unreacted reagents. Finally, the products were dried at 60 °C for 24 h for further use.

### 2.4. Adsorption Experiments

To investigate the binding capacity, 3 mg of Fe_3_O_4_@Au@PDA MNPs was incubated with 1.0 mL Cu(II) solution at different concentrations for an optimized time. After separation, the final Cu(II) concentration of the supernatant was determined by cyclic voltammetry and calculated by peak current. The amount of Cu(II) adsorbed by the Fe_3_O_4_@Au@PDA MNPs was calculated from the following formula [25,26]:

(1) $$Q=\frac{(C_{i}-C_{f})\times V}{m}$$

where:

*Q*—mass of Cu(II) adsorbed by unit mass of dry particles, mg/g
*C_i_*—Cu(II) concentrations of the initial solutions, mg/L
*C_f_*—Cu(II) concentrations of the final solutions, mg/L
*V*—total volume of the adsorption mixture, L
*m*—is the mass of the used particles, g

To investigate the effect of adsorption, the removal efficiency of ions is another evaluation parameter to study the adsorption properties of Fe_3_O_4_@Au@PDA MNPs. Removal efficiency of ions was calculated using the following formula [27,28]:

(2) $$\eta =\frac{C_{i}-C_{f}}{C_{i}}\times 100$$

where:

η—Removal efficiency, %
*C_i_*—ion concentration before treatment, mg/L
*C_f_*—ion concentration after treatment, mg/L

### 2.5. Selective Removal of Cu(II) from Water

Ag^+^, Mn^2+^, Mg^2+^, Fe^3+^, Ni^2+^, Cd^2+^ and Pb^2+^ were selected as interfering ions. The experimental procedure is in accordance with the above adsorption experiment at 5.0 mmol/L of initial concentration. The copper ion is replaced by other ions. During the experiment, the contents of these metal ions including Cu^2+^ which were diluted 50-fold were measured by flame atomic absorption spectrometry at WFX-120 (Beijing Rayleigh Analytical Instrument Co., Ltd., Beijing, China).

### 2.6. Electrochemical Characterization

The electrochemical analysis was performed with an electrochemical workstation (CHI 660D, Shanghai, China). A conventional three-electrode system was used, comprising a bare glassy carbon electrode(GCE) as working electrode, an Ag/AgCl electrode as reference electrode, a platinum wire as auxiliary electrode. The GCE was polished with 0.05 mm alumina slurry followed by sonicating and rinsing with water, then drying at room temperature. Cyclic voltammetry(CV) was carried out in 1-mL Cu(II) solutions (containing 100 μL supernatant, 1 mol/L KCl, pH = 2).

## 3. Results and Discussion

### 3.1. Synthetic Strategy of Magnetic Nanocomposites

**Figure 1:**
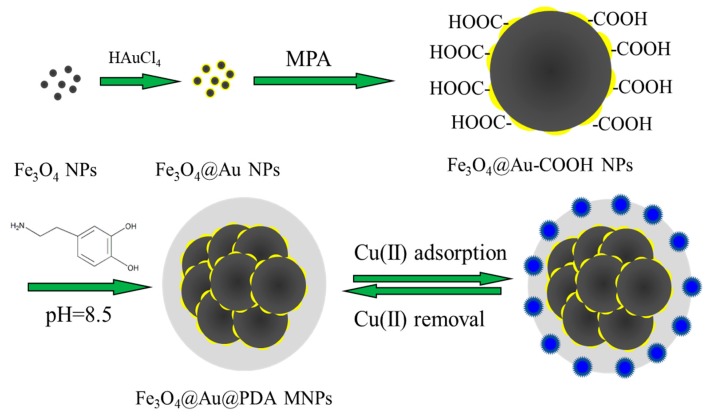
Synthesis route of magnetic nanocomposites.

In virtue of unique properties such as extraordinary biocompatibility, excellent dispersibility in aqueous phase, etc., surface modification on nanoparticles by PDA has been proved to be an effective method [29,30]. PDA with abundant active groups, especially catechol groups, can interact with metals ions through electrostatic, hydrogen bonding interactions or bidentate chelating. Therefore, PDA demonstrates potential applications in immobilization and separation of metal ions (e.g., Ti^4+^, Fe^3+^, Cu^2+^, Pb^2+^, Cd^2+^) [21,22,23,31]. In this manuscript, we found a novel magnetic nanocomposite with PDA that can effectively remove Cu(II). Herein, we prepared PDA-coated carboxyl functionalized Fe_3_O_4_@Au NPs for removal of Cu(II), The synthesis strategy is shown in Figure 1. Firstly, a chemical coprecipitation of Fe^2+^ and Fe^3+^ under sodium citrate media was adopted for the preparation of the Fe_3_O_4_ NPs. The sodium citrate dispersed Fe_3_O_4_ NPs favoured the formation of a hydrophilic core-shell Fe_3_O_4_@Au NPs which was prepared via in situ reduction of chloroauric acid. Secondly, the purpose of Au addition to Fe_3_O_4_ NPs was to protect Fe_3_O_4_ in a harsh environment and prevent oxidation of Fe(II). The uncoated gold nanoparticles can be removed by magnetic field separation. Furthermore, Au coating on Fe_3_O_4_ NPs is beneficial for surface functionalization by Au-S bonding. Through MPA functionalization on the surface of Fe_3_O_4_@Au NPs, carboxyl groups can be easily introduced to form electrostatic interactions with the amino group of dopamine. Moreover, the surface of Fe_3_O_4_@Au is easily wrapped with PDA because PDA is easy to deposit on the metal surface. Finally, the carboxyl functionalized Fe_3_O_4_@Au NPs were easily wrapped by a layer of PDA after being dispersed in dopamine solution under the alkaline environment (10 mmol/L Tris, pH = 8.5) which initiated polymerization of dopamine. The PDA layer can effectively adsorb Cu(II) with good selectivity and reproducibility.

### 3.2. Characterization of MNPs

**Figure 2:**
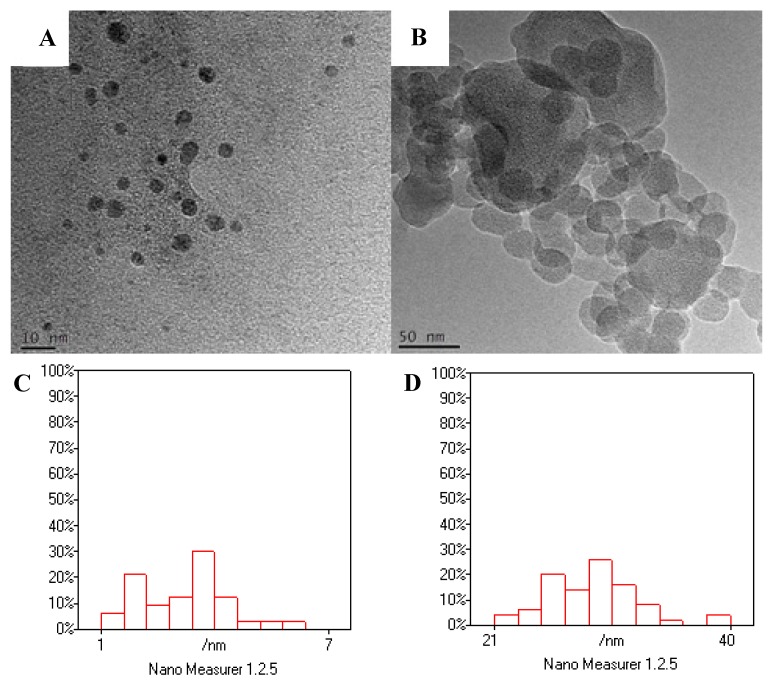
TEM images of Fe_3_O_4_ NPs(**A**); Fe_3_O_4_@Au@PDA MNPs (**B**); and particle size distribution diagram of Fe_3_O_4_ NPs (**C**) with total 33 particles; Fe_3_O_4_@Au@PDA MNPs (**D**) with total 50 particles by Nano Measurer 1.2.5. The *y*-axis % represents the proportion of nanoparticles in a certain size range.

The morphologies, structures, components and other physicochemical properties of the Fe_3_O_4_@Au@PDA MNPs were characterized by various techniques. The morphologies and structures of the MNPs were characterized by field emission transmission electron microscope (TEM, Tecnai F30 G^2^ 300 KV, Hillsboro, OR, USA). The TEM images of the MNPs are shown in Figure 2A,B to investigate morphological structures. As we can see from the figures, it is obvious that all of particles are nano-sized and roughly spherical in shape. Figure 2C shows that the diameter range of the Fe_3_O_4_ NPs is about 1.5~6.4 nm and the average diameter is 3.2 nm. After being wrapped by PDA, Figure 2B,D shows an average diameter about 29.1 nm of Fe_3_O_4_@Au@PDA MNPs with the range of 21.2~39.7 nm. From Figure 2B, the layer of PDA was visible, and no free Fe_3_O_4_ or Fe_3_O_4_@Au-COOH NPs were observed which indicated that magnetite NPs were successfully wrapped by PDA.

**Figure 3:**
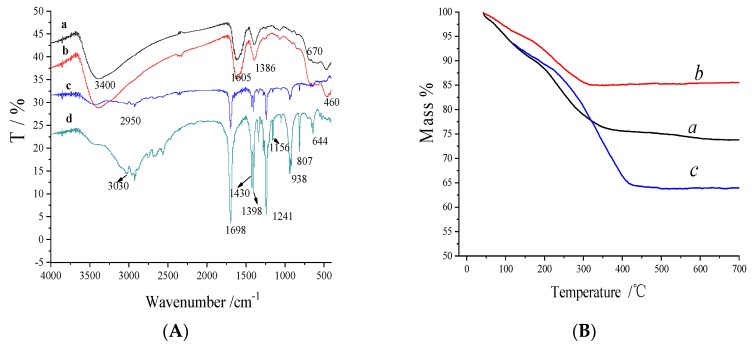
FTIR spectra (**A**) of Fe_3_O_4_ NPs (a), Fe_3_O_4_@Au NPs (b), Fe_3_O_4_@Au-COOH NPs (c), Fe_3_O_4_@Au@PDA MNPs (d), TGA curves (**B**) of Fe_3_O_4_ NPs (a), Fe_3_O_4_@Au NPs (b), Fe_3_O_4_@Au@PDA MNPs (c).

Fourier transform infrared spectroscopy (FT-IR, Nicolet, Madison, WI, USA) was employed to characterize the preparation procedure of Fe_3_O_4_@Au@PDA MNPs. As shown in Figure 3A, FT-IR spectra of Fe_3_O_4_ NPs (a), Fe_3_O_4_@Au NPs (b), Fe_3_O_4_@Au-COOH NPs (c), Fe_3_O_4_@Au@PDA MNPs (d), were compared. The peaks at 455 and 670 cm^−1^ in curves a–b were related to the Fe–O group, and the peak around 3400 cm^−1^ was assigned to the –OH vibrations on the surface of Fe_3_O_4_ NPs. Characteristic absorption peaks of Fe_3_O_4_ NPs decreased significantly in curve c and d, which means the surface of Fe_3_O_4_@Au NPs was modified. The absorption band at 1698 cm^−1^ corresponds to the carbonyl group of functionalized carboxyl groups (curve c and d). The absorption bands of 1430 to 1398 cm^−1^ related to –CH_2_ bending vibration. After being coated with PDA, many new infrared absorption peaks are generated in curve d. The broad and weak absorption bands near 3030 cm^−1^ originated from the benzene ring in PDA. The absorption peak at 1241 cm^−1^ contain a C-N stretching vibration. An Ar−H bending vibration (807 and 644 cm^−1^) was assigned to 1,2,4-substitued aromatic compounds. These results confirmed that Fe_3_O_4_@Au NPs had been successfully encapsulated by PDA via in situ oxidative polymerization.

Thermo-gravimetric analysis (TGA, STA 449C Netzsch, Bavaria, Germany) was used to determine the relative composition of the Fe_3_O_4_@Au@PDA MNPs. TGA was performed using dry powder samples with a heating rate of 10 °C/min up to 600 °C under a nitrogen atmosphere. As we can see from Figure 3B, the weight loss of Fe_3_O_4_ (curve a) and Fe_3_O_4_@Au NPs (curve b) from 100 to 600 °C was about 26% and 14%, respectively, which may be due to the loss of water and citrate ions on nanomaterial surface. Au coated on the Fe_3_O_4_ NPs leads to less weight loss. However, after being coated with PDA, Fe_3_O_4_@Au@PDA MNPs (curve c) show higher weight loss than Fe_3_O_4_ NPs and Fe_3_O_4_@Au NPs, which were about 36% mass percent. The result indicates that the content of the PDA coating was about 22% which further supported that PDA successfully wrapped the Fe_3_O_4_@Au NPs.

**Figure 4:**
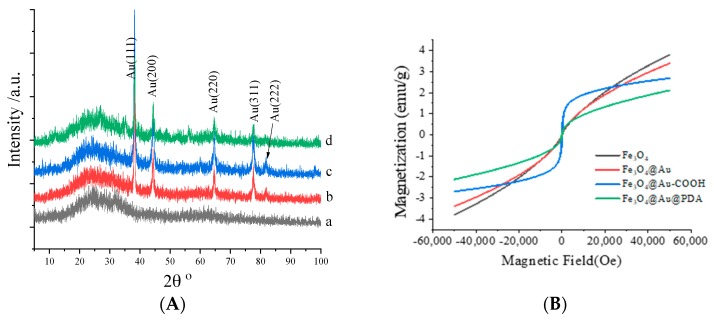
XRD spectra (**A**) and magnetization hysteresis loops (**B**) of the magnetic nanomaterials. Fe_3_O_4_ NPs (a), Fe_3_O_4_@Au NPs (b), Fe_3_O_4_@Au-COOH NPs (c), Fe_3_O_4_@Au@PDA MNPs (d).

The structure of magnetic nanomaterials was also characterized by X-ray diffraction (XRD, MiniFlex 600, Tokyo, Japan) as shown in Figure 4. No obvious diffraction peak in Figure 3a indicated the crystal was not produced which may be due to the low temperature of the nanomaterial treatment. After being wrapped by Au, a series of obvious diffraction peaks appeared. The peaks at 38.20, 44.31, 64.63, 77.64 and 81.85 in Figure 3b–d are ascribed to (111), (200), (220), (311) and (222) reflections of the Au face-centered cubic crystallographic structure (JCPDS card No. 65-2870). All the patterns illustrate that Au has been successfully loaded onto the Fe_3_O_4_ NPs. No change in the peak positions of Fe_3_O_4_, Fe_3_O_4_@Au NPs and Fe_3_O_4_@Au@PDA MNPs indicated that the surface modification has no effect on the Au crystal form.

Magnetization was detected with a LDJ9600 vibrating sample magnetometer (VSM, Troy, MI, USA) at ambient temperature. The vibrating sample magnetization (VSM) curves of Fe_3_O_4_ NPs, Fe_3_O_4_@Au NPs, Fe_3_O_4_@Au-COOH NPs, Fe_3_O_4_@Au@PDA MNPs are shown in Figure 4B. The superpara-magnetism nature of these materials can be proved by the hysteresis loops. All these materials have obvious magnetism. After wrapping with Au, the magnetism of Fe_3_O_4_@Au NPs decreased slightly. After cross-linking with COOH and PDA step by step, the saturation magnetization of Fe_3_O_4_@Au-COOH NPs, Fe_3_O_4_@Au@PDA MNPs decreases accordingly. However, it is still strong enough for magnetic isolation.

### 3.3. Electrochemical Detection of Cu(II)

**Figure 5:**
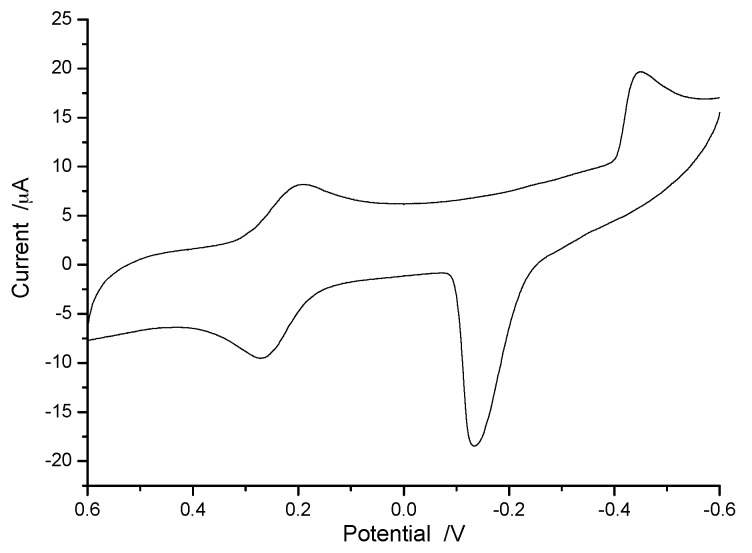
CV of 5.0 mmol/L Cu(II) in 0.1 mol/L HCl and 1.0 mol/L KCl, scan rate: 100 mV·s^−1^.

There are many methods to detect copper ions, such as fluorescent probes, atomic adsorption spectrophotometry, electrochemistry, etc. Among these methods, due to high sensitivity and easy operation, electrochemical analysis is the best analytic method. In our previous study [32], conditions of electrochemical detection to Cu(II) were optimized. We found that the peak current intensity of Cu(II) is largely affected by the electrolytes and pH in solution. According to the literature [32], a Cu(II) solution containing 0.01 mol/L HCl and 1.0 mol/L KCl was scanned by cyclic voltammograms (CV) using bare glassy carbon electrode with high signal response. Figure 5 shows the CV of 5.0 mmol/L Cu(II) solution containing 0.01 mol/L HCl and 1.0 mol/L KCl. Two pairs of irreversible redox peaks at 0.25 and −0.15 V can be clearly seen from Figure 5. Considering the convenience of detection, the peak potential at −0.15 V was selected for quantitative analysis.

### 3.4. Interaction of Magnetic Nanomaterials with Cu(II)

**Figure 6:**
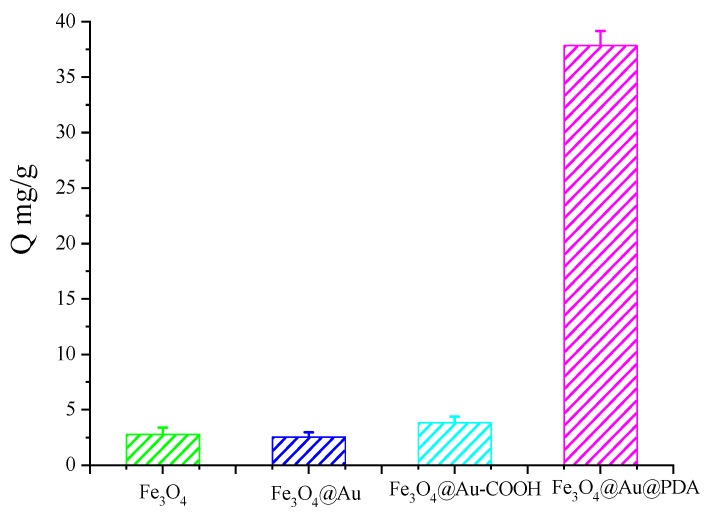
Adsorption mass of Cu(II) on different magnetic nanomaterials. *V* = 1.0 mL, *m* = 3.0 mg, *C_i_* = 10.0 mmol/L, time 2 h, temperature RT.

Nanomaterials may interact with molecules due to their nano-size and large surface-to-mass ratio. The adsorption properties of nanomaterials are highly affected not only by the weak intermolecular interaction, such as hydrophobic interactions, electrostatic interactions, hydrogen bonding, van der waals, but also by the intrinsic characteristics (e.g., charge, size, shape, electronic states, crystallinity, coatings, surface modifications with active groups, surface wrapping in the biological medium, hydrophobicity, and hydrophilicity). Therefore, we investigated the adsorption properties of Cu(II) on several kinds of magnetic nanomaterials. As shown in Figure 6, all the nanomaterials have a certain adsorption mass to Cu(II), but the Fe_3_O_4_@Au@PDA MNPs exhibit a distinct adsorption capacity compared to other magnetic nanomaterials, which indicates that Fe_3_O_4_@Au@PDA MNPs have a specific interaction with Cu(II).

### 3.5. Effects of DA Polymerization Time on Cu(II) Removal

**Figure 7:**
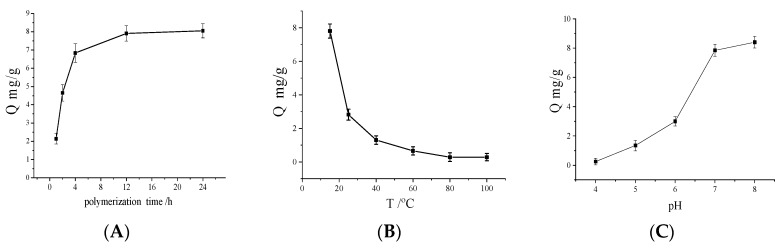
Adsorption mass of Cu(II) changed with DA polymerization time (**A**); temperature (**B**); pH (**C**). *V* = 1.0 mL, *m* = 3.0 mg, *C_i_* = 1.0 mmol/L, time 2 h.

Dopamine is a kind of biological neurotransmitter. In aqueous solution, it can be oxidized by dissolved oxygen and undergoes an oxidation crosslinking reaction, forming a composite layer of PDA that strongly attaches to a substrate. The PDA layer contains abundant catechol groups. The adsorption mass change with DA polymerization time was calculated (Figure 7A) to investigate the adsorption effect of Cu(II). As shown in Figure 7A, an increase in DA polymerization time brings about a significant increase in adsorption capacity which reaches a threshold corresponding to the best adsorption condition when the DA polymerization time is 12 h. Therefore, 12 h was selected as the optimized polymerization time.

### 3.6. Effects of Temperature on Cu(II) Adsorption

Metal ion absorption is often influenced by temperature. To investigate the influence of temperature, the adsorption behavior was examined in an aqueous medium at different temperatures. Figure 5B presents the effect of temperature on the adsorption capacity of Fe_3_O_4_@Au @PDA MNPs. As the temperature increased from 15 to 100 °C, the adsorption capacity first decreased dramatically, then declined slowly over 25 °C, and lost the adsorption performance completely by 80 °C. Results indicated that the high adsorption capacity of Fe_3_O_4_@Au@PDA MNPs on Cu(II) at a low temperature is due to the exothermic nature of the adsorption reaction [33]. Therefore, 15 °C was selected as the optimal adsorption temperature. At the same time, the adsorption of Cu(II) decreased dramatically from Fe_3_O_4_@Au@PDA MNPs, indicating that it is a physical interaction at low temperature.

### 3.7. Effects of pH on Cu(II) Adsorption

Solution pH is another important factor affecting the adsorption characteristics of the adsorbents due to the surface charges being largely influenced by the solution environment. To evaluate the effect of pH values on Cu(II) adsorption to Fe_3_O_4_@Au@PDA MNPs, we conducted a set of experiments in different pH solutions containing the same initial concentrations of the 1.0 mmol/L Cu(II) solution. Considering the stability of Cu(II) ion in acidic condition, pH values were adjusted from 4.0 to 7.0 (Figure 7C). From the result, it can be found that with increasing pH values, the surface charges of Fe_3_O_4_@Au@PDA MNPs became more negative, and the adsorption capacities of Cu(II) dramatically increased in the range of pH values from 4.0 to 7.0. When pH exceeded 7.0, with pH increasing, metal oxide is gradually formed, and produces precipitate. In this condition, the removal mechanism of metal ions will become complicated, and it will be difficult to distinguish between the adsorption and precipitation of metal ions. Therefore, 7.0 was selected as the optimized solution pH. Generally, materials for removing copper require an environment of pH > 7; the pH needs to be adjusted repeatedly, and a waste alkali. pH of 7.0 can simplify the Cu(II) adsorption process and be conducive to industrial application.

### 3.8. Adsorption Kinetic Studies

**Figure 8:**
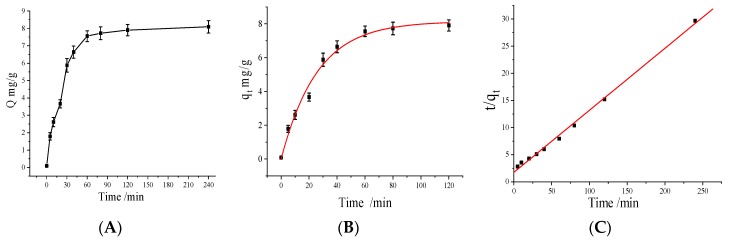
Effect of time (**A**), pseudo-first-order (**B**) and pseudo-second-order (**C**) kinetic models for Cu(II) adsorption. *V* = 1.0 mL, *m* = 3.0 mg, *C_i_* = 1.0 mmol/L, temperature 15 °C, pH 7.0, time 2 h.

Adsorption kinetics describe the solute uptake rate which in turn controls the residence time of adsorbate uptake at the solid-solution interface. Therefore, the kinetics can provide valuable insights into the mechanism and reaction pathway of adsorption process [34,35]. To gain further insight into the adsorption mechanism of MNPs, adsorption kinetics were investigated. The adsorption tests are carried out in a Cu(II) solution with 1.0 mmol/L at pH 7.0. The effect of contact time on the adsorption of the Fe_3_O_4_@Au@ PDA MNPs for Cu(II) ions is shown in Figure 8A. As can be seen, the adsorption occurs rapidly in the first 30 min, and then the adsorption rate slows down. Finally, the adsorption capacity achieves a state of equilibrium after 2 h. It is found that the adsorption capacity of the Fe_3_O_4_@Au@ PDA MNPs reaches 7.90 mg/g.

The pseudo-first order and the pseudo-second order kinetic models are used to simulate the adsorption kinetics of the Fe_3_O_4_@Au@ PDA MNPs for Cu(II) ions. These two rate equations are shown below. The pseudo-first order kinetic model suggested by Lagergren for the adsorption of solid/liquid systems can be expressed as [36]:

*q_t_* = *q_e_* [1 − *e*^(−*k*_1_*t*)^] (3)

Ho and McKay’s pseudo-second order kinetic model can be expressed as:

(4) $$\frac{t}{q_{t}}=\frac{t}{q_{e}}+\frac{1}{k_{2}{q_{e}}^{2}}$$

where *k*_1_ is the Lagergren rate constant of adsorption (min^−1^), *k*_2_ is the pseudo-second-order rate constant of adsorption (g·mg^−1^·min^−1^). *q_e_* and *q_t_* are the amounts of Cu(II) adsorbed (mg·g^−1^) at equilibrium and at time *t*, respectively. The values of *k*_1_, *k*_2_ and the correlation coefficient (*R*) can be determined experimentally by plotting *q_t_* versus t and *t/q_t_* versus t, respectively.

A plot (Figure 8B) of *q_t_* versus *t* according to the pseudo-first-order kinetic model gives a fitting curve in the initial 120 min. The correspondence with the pseudo-first-order kinetic model substantiates that Cu(II) adsorption onto the Fe_3_O_4_@Au@ PDA MNPs is a diffusion-based process. However, the pseudo-second-order kinetic model (Figure 8C) is suitable for the whole adsorption process, which indicates that the adsorption of Cu(II) onto the Fe_3_O_4_@Au@ PDA MNPs is controlled by chemical adsorption. The kinetic parameters of the Fe_3_O_4_@Au@ PDA MNPs calculated from Equations (3) and (4) are listed in Table 1, which shows that the value of *R* (*R* > 0.99) was high, suggesting that both models are well fitted to the experimental results. Therefore, the adsorption process can essentially be divided into two steps. The first step is mass transfer through a water film to the adsorbent surface (film diffusion) in the initial 80 min; the second one is occupation at a site on the surface through chemical adsorption over 80 min [37].

### 3.9. Adsorption Isotherms

**Figure 9:**
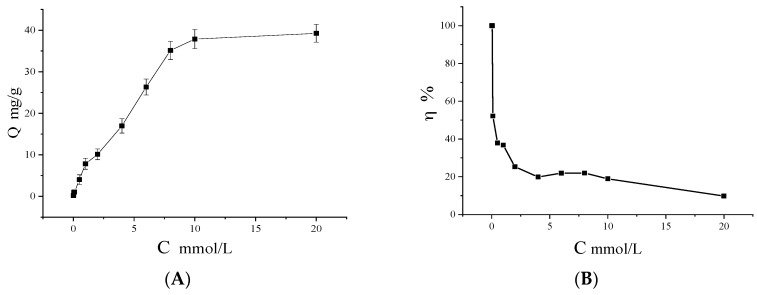
Adsorption isotherms (**A**) and removal rate (**B**) of Cu(II) on the MNPs. Adsorption conditions: *V* = 1.0 mL, *m* = 3.0 mg, *C_i_* = 0–20 mmol/L, time 2 h, temperature 15 °C, pH 7.0.

Adsorption isotherms describe how the process of adsorption proceeds on the adsorbent surface [38]. The adsorption isotherm experiments for the prepared MNPs were carried out at different initial concentrations of Cu(II), ranging from 0 to 20 mmol/L. As shown in Figure 9A, it was observed that the adsorption amount of Cu(II) ions on MNPs rapidly increased with the increase of Cu(II) concentration from 0 to 10 mmol/L, and reached equilibrium over 10 mmol/L. In this case, a high saturated adsorption capacity of 37.86 mg/g was obtained when the Cu(II) concentration is 10 mmol/L. As shown in Figure 9B, the removal efficiency of Cu(II) reached 100% when the Cu(II) concentration was below 0.05 mmol/L, which meets the standard for purified drinking water, and then rapidly decreased with the increase in the Cu(II) concentration. When the concentration is 1.0 mmol/L, the removal rate can still reach 36%, which shows good removal efficiency for Cu(II) in a water environment. An increase in the removal rate for high concentrations of Cu(II) can be achieved by increasing the amount of adsorbent and with the repeated use of adsorbents.

The theoretical adsorption capacity of MNPs can be described by Langmuir and Freundlich equations. The Langmuir model is a model that assumes monolayer coverage of a finite number of identical sites present on the surface such that no further adsorption takes place [39]. The Freundlich model describes non-ideal and reversible adsorption, not limited to monolayer formation. It can be applied to multilayer adsorption, with non-uniform distribution of adsorption heat and affinities over a heterogeneous surface [40]. The Langmuir and Freundlich equations are expressed as follows:

Langmuir equations:

(5) $$\frac{c_{e}}{q_{e}}=\frac{c_{e}}{q_{m}}+\frac{1}{q_{m}k_{L}}$$

Freundlich equation:

(6) $$Inq_{e}=Ink_{F}+\frac{Inc_{e}}{n}$$

where *c_e_* (mg/mL) is the equilibrium concentration of Cu(II) ions, *q_e_* (mg/g) is the adsorption capacity, *q_m_* (mg/g) is the theoretical saturation adsorption capacity, *k_L_* is the Langmuir constant, *k_F_* is the binding energy constant and n is the Freundlich constant.

**Figure 10:**
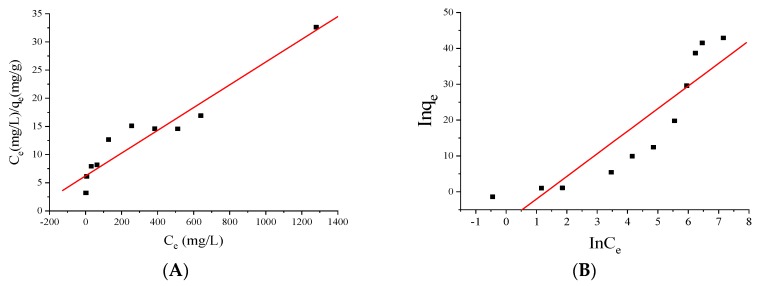
The linear fitting curves of the Langmuir (**A**) and Freundlich (**B**) models.

The linear fitting curves of the Langmuir and Freundlich models are shown in Figure 10A,B, respectively. As we can see, according to the values of correlation coefficients (*r*_Langmuir_ = 0.95678, *r*_Freundlich_ = 0.90058), the Langmuir model gave a better fit, indicating that the adsorption of Cu(II) ions on MNPs is homogeneous adsorption on the surface of MNPs.

### 3.10. Selective Adsorption of Cu(II) Ions

**Figure 11:**
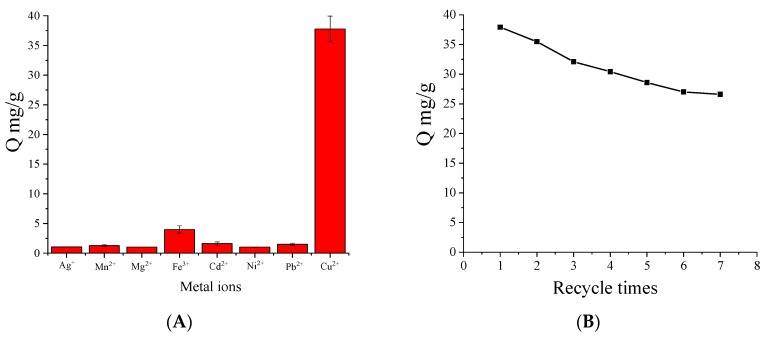
Adsorption of MNPs toward metal ions (**A**) and effect of regenerative times on the adsorption capacity (**B**). Adsorption conditions: *V* = 1.0 mL, *m* = 3.0 mg, *C_i_* = 10.0 mmol/L, time 2 h, temperature 15 °C, pH 7.0.

Selectivity is another index to evaluate the performance of an adsorbent. In general, the metal-ion sorbents have a good adsorption capacity for a certain kind of ions, such as heavy metal ions [41], (Cu^2+^, Ag^+^, and Hg^2+^) [42], (Cr^5+^ and Cu^2+^) [43], but the selective adsorption of copper ion has not discussed in depth. Chouyyok et al. reported that a kind of nanoporous sorbent functionalized with chelating diamines had excellent selectivity for Cu^2+^ over other metal ions (e.g., Ca^2+^, Fe^2+^, Ni^2+^, and Zn^2+^) [44]. In order to investigate the selective adsorption capacity of MNPs on different metal ions, some metal ions were selected as interfering ions, including Ag^+^, Mn^2+^, Mg^2+^, Fe^3+^, Cd^2+^, Ni^2+^, Pb^2+^ and Cu^2+^. As shown in Figure 11A, it was observed that the adsorption amount of Cu(II) ions on MNPs is significantly higher than for other metal ions. In particular, the MNPs have almost no adsorption to Ag^+^, Mn^2+^, Ni^2+^ and a weak adsorption to Fe^3+^, Cd^2+^, Pb^2+^. This result confirms that the proposed MNPs showed good selectivity to these interfering metal ions.

### 3.11. Regeneration Studies

To keep the processing cost down and for potential practical application, it is preferable to examine the possibility of desorbing Cu(II) ions from MNPs for its reuse. A desorption experiment was carried out by controlling the temperature above 60 °C. Seven adsorption-desorption consecutive cycles were performed to evaluate the reuse possibility of MNPs for Cu(II) adsorption. As shown in Figure 11B, the regenerative MNPs still possessed a high adsorption capability, which declined slightly with increasing cycle times. The adsorption capacity decreased to 5.59 mg/g (about 70% of the initial value) after five regeneration periods and 4.60 mg/g (about 58% of the initial value) after five regeneration periods. Result confirmed the good reusability and stability of the adsorbent. Regeneration studies give better results than Khan Rao’s three cycles [45] and Wu’s five cycles [46].

## 4. Conclusions

In this study, novel magnetic Fe_3_O_4_@Au@PDA nanocomposites were synthesized which can effectively adsorb Cu(II). Through high sensitive electrochemical monitoring, the adsorption performance of the MNPs was found to be greatly dependent on temperature, solution pH and initial Cu(II) concentration. The excellent adsorption behaviors were dominated by rich catechol groups of polydopamine. In addition, MNPs can be easily desorbed and repeatedly used by controlling the temperature above 60 °C. Furthermore, the as-prepared MNPs shows a good selectivity for removal Cu(II). Results indicate that the MNPs are efficient and environmentally friendly adsorbents for the selective removal of Cu(II) in aqueous solutions. 

## Figures and Tables

**Table 1:** Adsorption kinetic parameters for Cu(II) adsorption on MNPs.

	*Q_e_*, exp	*k*	*Q_e_*, cal	*R*
Pseudo-first-order	7.90	0.044	8.01	0.99942
Pseudo-second-order		0.0074	8.76	0.99839
